# Supplementary figures and images for: Characterization of Monoamine Oxidase-A in tropical liver fluke, Fasciola gigantica
Source: PLoS One. 2023 Apr 27;18(4):e0284991. doi: 10.1371/journal.pone.0284991 (PMC10138849; doi:10.1371/journal.pone.0284991)

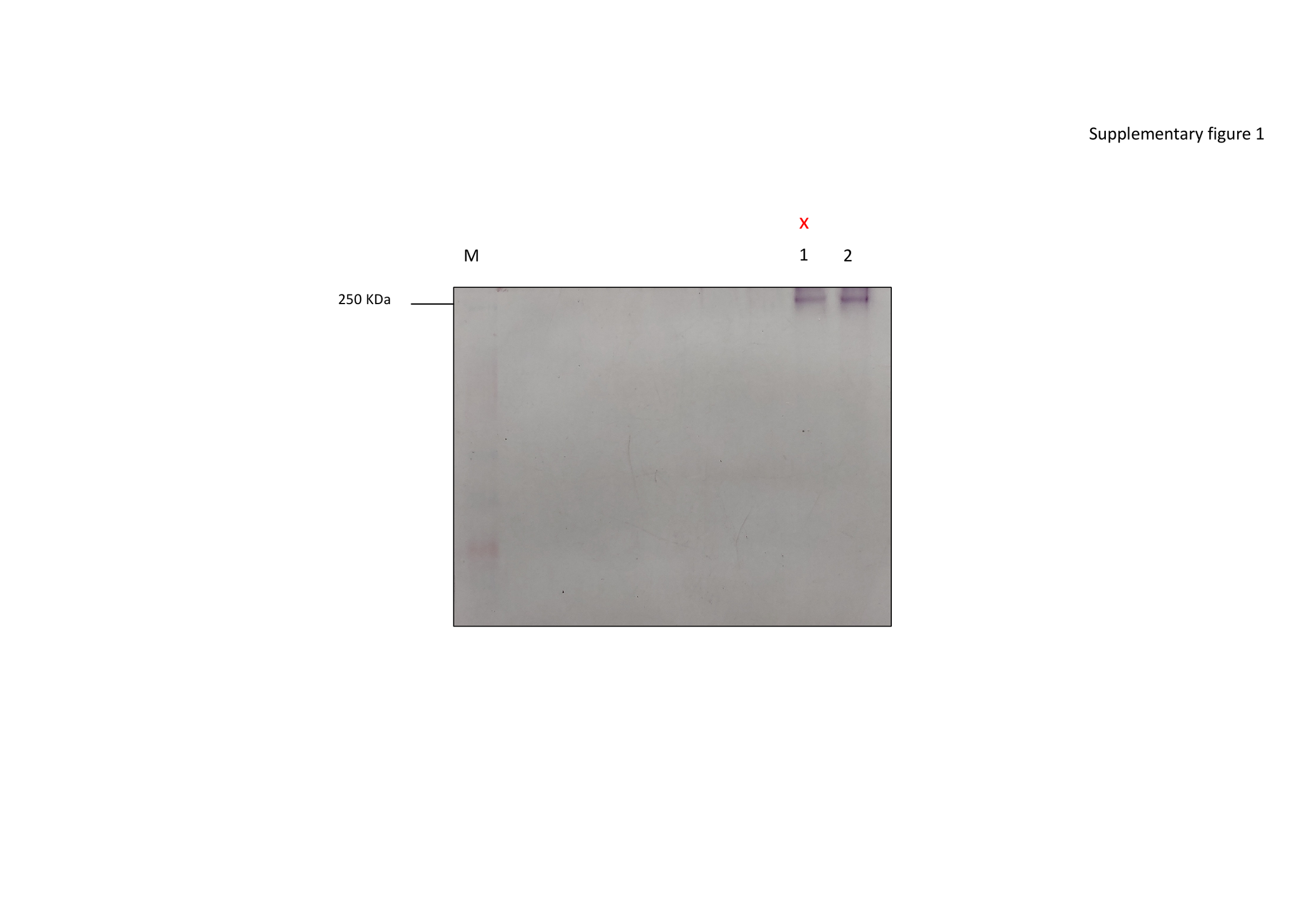

Supplement: S1 Fig — Note: Lane 1 is not the part of main figure and Marker in all zymography gels appeared to be very faint. (TIF) [file pone.0284991.s001.tif]

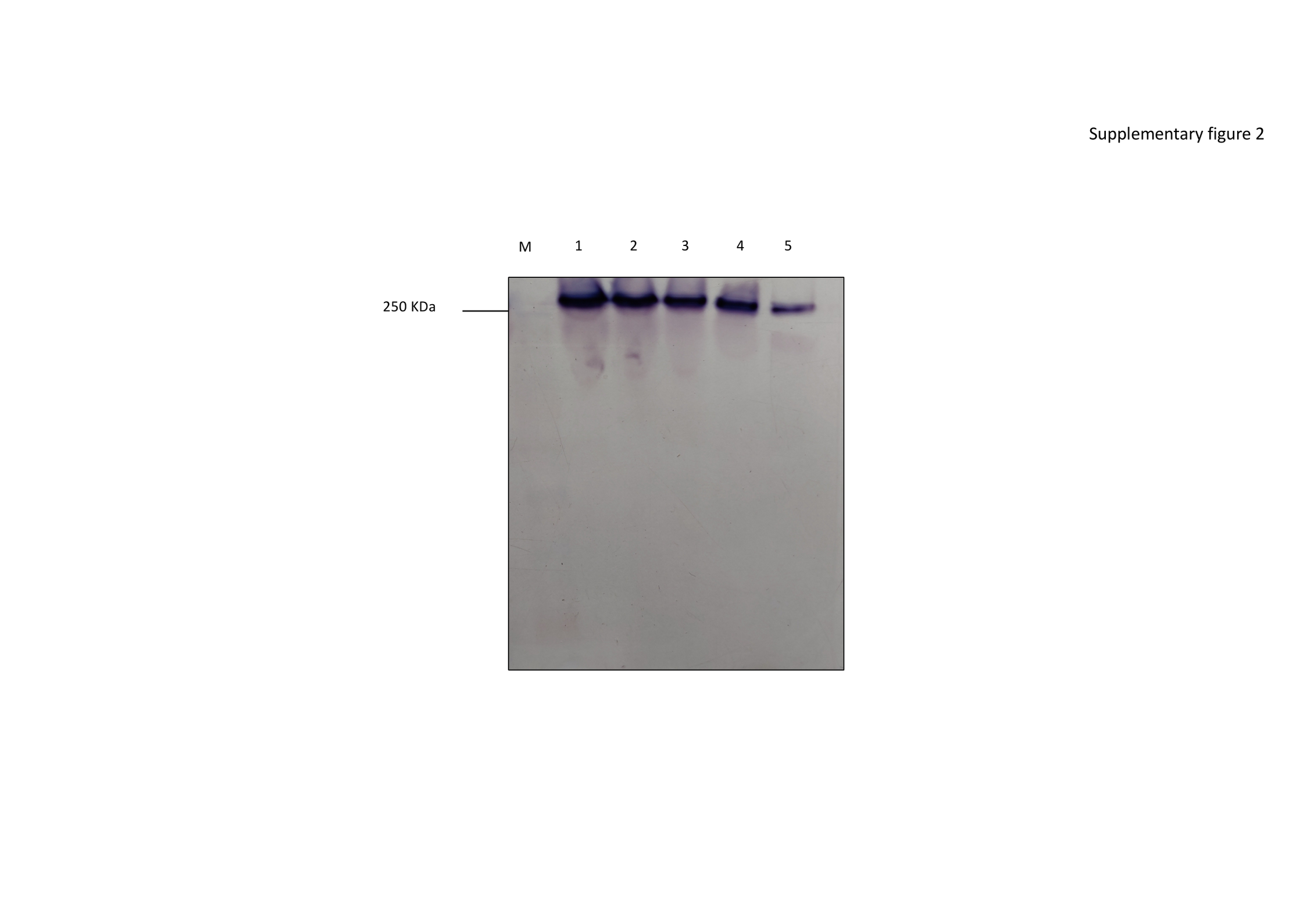

Supplement: S2 Fig — Note: Marker in all zymography gels appeared to be very faint. (TIF) [file pone.0284991.s002.tif]

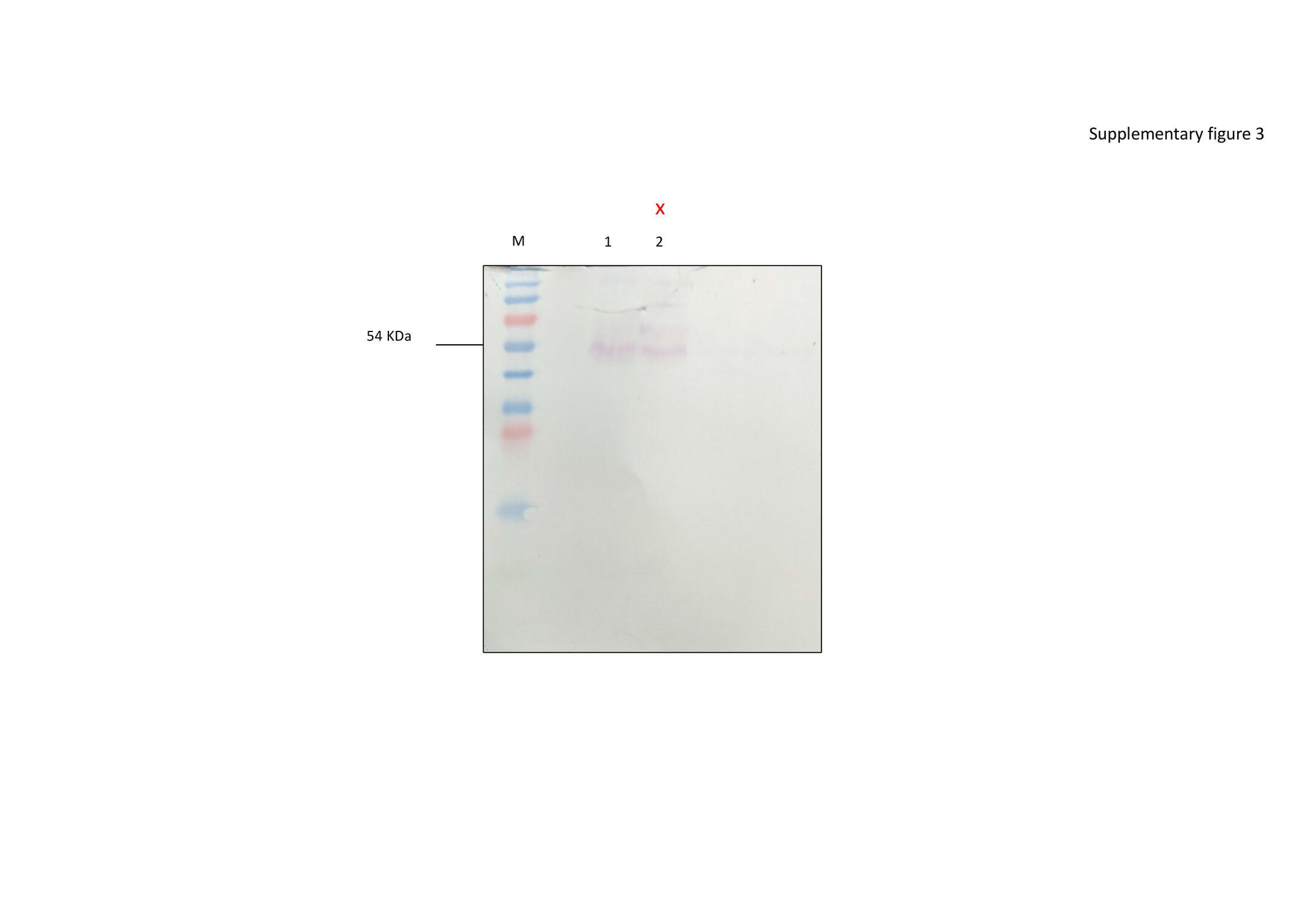

Supplement: S3 Fig — Note: Lane 2 is not the part of main figure. (TIF) [file pone.0284991.s003.tif]
