# Supplementary material for: Characterization of Monoamine Oxidase-A in tropical liver fluke, Fasciola gigantica
Source: PLoS One. 2023 Apr 27;18(4):e0284991. doi: 10.1371/journal.pone.0284991 (PMC10138849; doi:10.1371/journal.pone.0284991)
Supplement: Attachment — Submitted filename: Minor Revision PONE-D-22-30479.pdf [file pone.0284991.s004.pdf]

PONE-D-22-30479

Title: "*Characterization of Monoamine Oxidase-A in tropical liver fluke, Fasciola gigantica.*"

Minor Revision

The publication does not overstate anything and addresses the titular claims well. It is a well referenced manuscript and authors have nicely mentioned drug resistance story and explained the present gaps in the characterization of MAO in helminths. Furthermore, characterization through multi-omics approach validates most of the findings. I believe molecular characterisation of parasite is a strong pivot of this manuscript because identification of field-procured parasite just on the basis of morphological identification could give false positive results. Although, I have no doubt on experimentation but the quality of manuscript can be improvised by small changes which I have enumerated below:

1. It is mentioned that MAO activity in mitochondria is significantly higher compared to whole homogenate but there is no significant asterisks on activity bars. Kindly put significant marks in Fig 2a.

2. Authors have forgotten to put lane 2 western blot in Fig 4b. Kindly fix that.

3. In Abstract, abbreviate the full form of 'WHO' as 'World Health Organization' because it is not a good idea to introduce abbreviated words in abstract because it is the first text the audience reads.

4. Almost at all the places spelling of 'Dependent' is wrong. Kindly replace "Dependent" with "Dependant". For spellings, stay with either UK English or US English in a manuscript and not a mixture of both in a single publication.

5. Avoid using same adjective in a single sentence such as in this statement "The strong intensity of bands in western blots and the spots in Dot-blots indicate strong immunogenic nature of the MAO protein" strong is used two times as an adjective. Then again after two three lines "clearly indicating that the tropical liver fluke possesses strong MAO-A activity" the use of word "strong" comes into play. Kindly replace it with different synonyms.

6. The statement in 3rd paragraph of introduction "thus making MAO an indispensable biomolecule for developing the host-parasite interaction for the survival and perpetuation" does not make any sense or it is too complex. Kindly use simpler text for a better understanding for a wider range of audience.

7. Kindly rephrase the last paragraph statement in introduction “Previously researchers have reported and localized MAO in helminth parasites but data is still in dearth about the complete picture of this enzyme in a specific parasite”.

8. In the conclusive remarks “Considering the present results, it can be elucidated that MAO-A is an important neurophysiological enzyme having a high molecular mass and is abundantly present in the mitochondria of tropical liver fluke, F. gigantica” refrain from using unspecific words such as “high molecular mass” instead of that mention the size of band observed.
