# Supplementary material for: Characterization of Monoamine Oxidase-A in tropical liver fluke, Fasciola gigantica
Source: PLoS One. 2023 Apr 27;18(4):e0284991. doi: 10.1371/journal.pone.0284991 (PMC10138849; doi:10.1371/journal.pone.0284991)
Supplement: Attachment — Submitted filename: Response to reviewers.docx [file pone.0284991.s005.docx]

First and foremost, we sincerely thank the reviewers for putting up their valuable time in reviewing this manuscript. We thoroughly went through the comments made by reviewers and have corrected our manuscript as advised. The corrections and explanations behind those changes are enumerated as follows:

Reviewer#1

**Query**: **Reviewer requested to include PCR reaction volume and concentrations in text.**

Response: PCR reaction volume and concentrations of each of its ingredient are now provided in materials and methods section (Page 6 under heading “Amplification of DNA using Polymerase Chain Reaction”).

**Query: Reviewer questioned about the statistical test done, if any, in figure 2b.**

Response: The non-parametric t-test was performed for figure 2a and One-way ANOVA was performed for figure 2b. The level of significance along with their significance asterisk have now been included in the graphs as advised (Figure 2).

**Query: Reviewer had advised to provide details about host of antibody and specificity of epitopes of antibody**.

Response: It is not possible to raise antibody in *Fasciola gigantica* because it does not have well defined circulatory system. The antibody was purchased from Sigma-Aldrich which was raised in mouse with the epitopes of human MAO and cited reactivity with mouse samples. Since the MAO is highly conserved protein and with its reactivity with mouse we hypothesized it should react with Fasciola samples too. No antibody of MAO against any parasite was available in the market.

**Query: Reviewer had requested to mention specificity of primers and size of amplicons obtained in ITS2 based molecular identification experiment. He had also advised to include positive and negative control in PCR.**

Response: As suggested, we have included negative control of PCR in our results. Since the experiment was done for the first time it was not possible to have positive control. Regarding the size of amplicon, it is in consensus with previously reported *Fasciola gigantica* ITS2 amplicons (300-500 bp) which were used by other researchers for molecular characterization of *F. gigantica* in their area of study. The primers used were specific for *Fasciola gigantica* the details of which is included in materials and methods section (Page 6 under the heading Amplification of DNA using Polymerase Chain Reaction).

**Query: Reviewer sought an explanation on successful extraction of mitochondria.**

Response: Here, we were not proving that MAO-A is localized in mitochondria. It is already an established fact that MAO-A is present in mitochondria and the substrate we used in our study for detection was specific for MAO-A enzyme which clearly explains why we saw significantly higher signals in mitochondrial extracts compared to whole homogenate.

Significantly high activity of MAO-A in mitochondrial extract compared to whole homogenate samples in itself shows that there was successful extraction of mitochondria which we did following well-proven method of Podesta et al. [50].

**Query: Reviewer had asked authors to include number of studies made in response to the statement “For the immunological characterization of MAO-A in *F. gigantica* worms a number of studies were performed”.**

Response: By this statement "For the immunological characterization of MAO-A in *F. gigantica* worms a number of studies were performed" we were not referring to the number of replicates. Our intent was to say that we adopted multipronged and multi-omics approach for the characterization of MAO such as biochemical, immunological and pharmacological for a profound validation of results.

**Query: Reviewer pointed out that there is no lane 2 in figure 4b.**

Response: We realised that legend of figure 4b was wrongly written and the legend is now corrected as suggested (Page 14, caption of Fig 4).

**Query: Reviewer had asked about loading control in clorgyline treated western blotting result.**

Response: Reviewer has raised a valid point of loading control but since the authors involved in this study have mov­­ed out of that lab it is not possible to replicate this experiment. Therefore, we have removed the western blot of clorgyline treated samples (Figure 7).

**Query: Reviewer requested to change “Till now” to “Until now”.**

Response: In the phrase " Till now, MAO has been reported in parasitic helminths belonging to different classes, namely ", till is changed to until (Page 5, second-last paragraph).

Reviewer#2

**Query: Reviewer had requested to put level of significance in Figure 2.**

Response: Asterisks for level of significance following statistical tests has now been added to the graphs (Fig 2).

**Query: Reviewer pointed out that legend and figure 3b is not in accordance with each other.**

Response: The legend and figure are now corrected and lane2 is removed from legend (Fig 3).

**Query: Reviewer had requested to write full form of WHO in abstract.**

Response: WHO has now been abbreviated in full as World Health Organisation in Abstract (Page 1).

**Query: Reviewer had asked to correct spelling of Dependant at all places in text.**

Response: The spelling of Dependent has now been changed to Dependant at all places (Page 2, 4, 15, 16 and 21).

**Query: Reviewer had pointed out some grammatical errors in Abstract and advised to correct it.**

Response: The repetition of adjectives has been replaced as advised (Page 2, last para).

**Query: Reviewer had requested to make changes to statement “thus making MAO an indispensable biomolecule for developing the host-parasite interaction for the survival and perpetuation”**

Response: The text “thus making MAO an indispensable biomolecule for developing the host-parasite interaction for the survival and perpetuation” has been reframed to make more sense (Page 4, para 1).

**Query: Reviewer had asked authors to correct the statement “Previously researchers have reported and localized MAO in helminth parasites but data is still in dearth about the complete picture of this enzyme in a specific parasite”.**

Response: The sentence “Previously researchers have reported and localized MAO in helminth parasites but data is still in dearth about the complete picture of this enzyme in a specific parasite” has been reframed as suggested (page 5, last para).

**Query: Reviewer had requested to include size of band in conclusion instead of high molecular mass.**

Response: In conclusion section, we have mentioned size of band as requested (Page 23).

The authors would like to sincerely thank the reviewers for their valuable inputs in making

our manuscript better. We hope that the revised version of the manuscript could now be

accepted for publication in Plos One journal.
